# Mystical Medical Politics

**Published:** 1860-02

**Authors:** 


### MYSTICAL MEDICAL POLITICS.

"The editor of the Chicago *Medical Journal*, speaking of the recent stampede of the Southern students from Philadelphia, says, 'if a *political* movement, he should regret it. But if, on the contrary, it was the result of a returning sense of the *mistake* the young gentlemen committed in passing by the schools of their own states, *especially for those of Pennsylvania*, he cannot but commend their judgment.'

"What do the words we have italicized mean? Would the 'mistake' have been less if the young gentlemen had passed by the schools of their own states for the Rush Medical College at Chicago?

"Our confrere is not alone in his wish for a more equal distribution of students, nor in his objections to five hundred being gathered on the seats of a single lecture room, as at Philadelphia. So, also, there are many of the teaching brethren who will join him in 'the hope that hereafter the medical students of the *West* will not be behind those in the *South* in their attachment to the institutions of their own particular *sections!*' But we are sorry to see sectionalism invoked in this connection. It is as bad as 'Helper's book,' which forbids employing Southern physicians. If this be not political, it would puzzle Dr. Brainard to find it. But Philadelphia will survive it; and we see no other way to have it otherwise, than to make a better school in Illinois than they have in Philadelphia."

We extract the above paragraph from a recent number of the *American Medical Gazette*, edited by Dr. D. M. Reese, on account of the fairness and independence which generally characterize that journal. If the editor had transferred the whole of our article it could not have been supposed that we intended to discriminate against Philadelphia schools.

There is nothing mystical about our views of the matter of medical teaching. We are of the opinion that many students resort to Philadelphia from the regions of St. Louis, Cincinnati, New Orleans, and perhaps New York and Boston, who would be as well intructed in these cities respectively, as they can be in Philadelphia. We look upon such students as making a mistake, and acting under erroneous impressions of the relative advantages of these cities.

If students left the state of Pennsylvania and adjoining states to assemble in classes of four or five hundred each, in New York, we should think the same of their conduct.

We may be mistaken in the relative value of the medical instruction in the cities named, for we have not visited all of them. But until we can see some evidence of this, we adhere to the opinion before expressed, that it is better for students, the profession, and the public, that medical schools should, when well organized and possessed of ample means of instruction, be sustained by patronage of the divisions of the country in which they are situated, and we shall labor to make one school deserving the support of the population of this region.

